# Charge Regulation in Liquid Films Stabilized by Ionic Surfactants: Change in Adsorption with Film Thickness and Phase Transitions

**DOI:** 10.3390/molecules30030659

**Published:** 2025-02-01

**Authors:** Iglika M. Dimitrova, Radomir I. Slavchov

**Affiliations:** 1 Department of Physical Chemistry, University of Chemical Technology and Metallurgy, Kliment Ohridski Blvd. 8, 1756 Sofia, Bulgaria; imd@uctm.edu; 2 Rostislaw Kaischew Institute of Physical Chemistry, Bulgarian Academy of Sciences, Acad. G. Bonchev Str., Bl. 11, 1113 Sofia, Bulgaria; 3 School of Engineering and Materials Science, Queen Mary University of London, Mile End Road, London E1 4NS, UK

**Keywords:** charge regulation, foam, emulsion, surface forces, adsorption, ionic surfactants, surface phase transition

## Abstract

When a liquid film is thinning, the charge and the potential of its surfaces change simultaneously due to the interaction between the two surfaces. This phenomenon is an example for charge regulation and has been known for half a century for systems featuring aqueous solutions in contact with metals, salts, biological surfaces covered by protolytes, etc. Few studies, however, investigated regulation in foam and emulsion films, where the charge is carried by soluble ionic surfactants. This work presents an analysis of the phenomenon for surfactants that follow the classical Davies adsorption isotherm. The electrostatic disjoining pressure *Π*_el_ was analyzed, and the Davies isotherm was shown to lead to *Π*_el_ ∝ *h*^−1/2^ behavior at a small film thickness *h*. As usual, the charge regulation regime (constant chemical potential of the surfactant) corresponded to a dependence of *Π*_el_ on *h* between those for constant charge and constant electric potential regimes. The role of the background electrolyte was also studied. At the water–air interface, many ionic surfactants exhibit a surface phase transition. We show that the interaction between the two surfaces of a foam film can trigger the phase transition (i.e., the film changes its charge abruptly), and two films of different *h* values can coexist in equilibrium with each other—one covered by surfactant in the 2D gaseous state and another in the 2D liquid state.

## 1. Introduction

All characteristics of an interface (adsorption, interfacial tension, surface potential, etc.) depend on the geometry of the heterogeneous system [1]. This dependence cannot be neglected whenever the characteristic size of the heterogeneity (the radius of curvature *R*, the thin film thickness *h*, etc.) is small compared with the characteristic thickness of the surface layer—which, for charged surfaces, is the Debye length *L*_D_. Therefore, an ionic surfactant does not adsorb to the same extent on a flat surface, on nanometer-sized droplets or on extremely thin foam films. In the context of surface forces, the effect of the confinement on the state of the surface is known as *charge regulation*. As opposed to the assumptions for a constant surface charge or surface potential [2], in a thin film, both the charge and potential change with *h*, and the films drain under a constant chemical potential.

The chemical potential of an adsorbed species is fixed to its bulk chemical potential, and this equality sets the adsorption isotherm (the *charge regulation condition*). The charge regulation condition is system-specific. For example, Bierman used the Langmuir isotherm [3] for the ion in the adsorption layer to provide the charge regulation condition. Later, several groups assumed that only the ions in the Stern layer are charge-regulated (while the surface itself is of a fixed charge), and they used the Stern [4,5,6,7,8] or Stern–Grahame model [9,10] for the description of the constant chemical potential regime. Variants of these theories were used for a range of surfaces. For example, Popa et al. [11] used the approach from [7] for oppositely charged latex particles, and Carnie and Chan [12] investigated solid surfaces covered with dissociable groups using the mass action law (which resulted in a Langmuir-type isotherm). Charge regulation is not limited to films; it plays its role in confined systems of other geometries, such as nanochannels [13], ion channels [14], nano-particles [15] and macro-ions [16].

Experimental investigations focusing on charge regulation have been performed for solid-liquid-solid surface forces using the surface forces apparatus [17] and the colloidal probe technique [18,19]. For foam films, the disjoining pressure *Π* is accessible via modifications to the Sheludko cell [20]. Emulsion films can also be investigated [21,22]. However, no dedicated study on charge regulation in fluid films is known to us.

This work sets the fundamental theory for charge regulation in foam and emulsion films stabilized by soluble ionic surfactants, and it is a necessary first step before a subsequent experimental study on the phenomenon. We study two cases. The first one is simpler, involving an oil–water–oil emulsion film stabilized by a water-soluble ionic surfactant which follows the Davies adsorption isotherm without phase transition, suitable for water–oil (W|O) interfaces. The Van der Waals attraction is negligible for emulsion films. The second case is a foam film, where the two monolayers of the ionic surfactant exhibit a surface phase transition, which is typical for water–gas (W|G) interfaces. The two surface phases follow two different Davies isotherms. For foam films, the Van der Waals attraction is significant.

## 2. Results and Discussion

### 2.1. The Poisson–Boltzmann Equation

**Figure 1:**
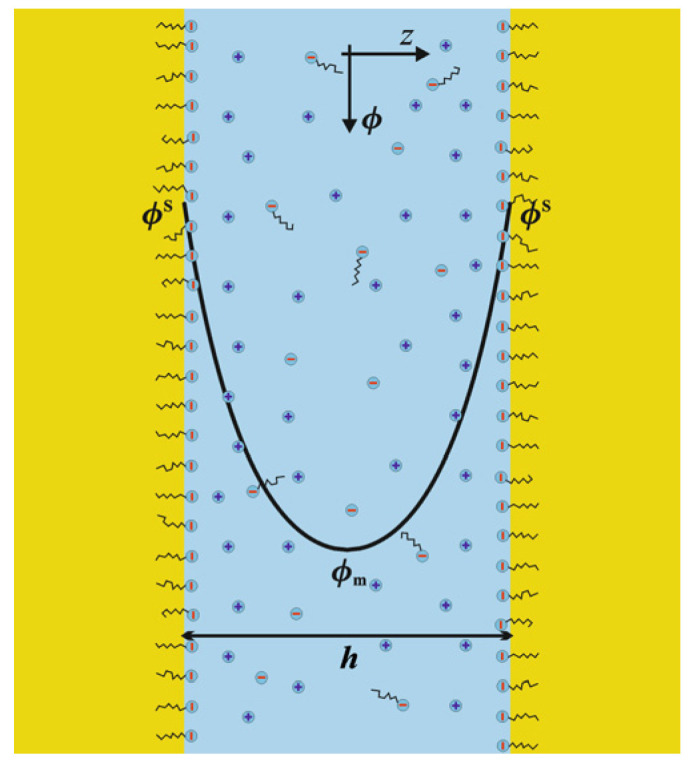
Illustration of thin oil–water–oil liquid film stabilized with ionic surfactant with the potential profile.

The electrostatic potential in the film is assumed to be the solution to the Poisson–Boltzmann equation:

(1) $$\varepsilon d^{2}\phi /dz^{2}=-\rho ,$$

where the bulk charge density *ρ* follows the Boltzmann distribution:

(2) $$\rho =eC_{\mathrm{el}}\left(\Phi -\Phi^{-1}\right);$$

here, *Φ* = exp(−*eϕ*/*kT*) is the Boltzmann factor for a cation; *e* is the charge of the ions, *ϕ* is the electrostatic potential, *z* is the distance from the center of the film (a symmetric film is considered; see Figure 1), *ε* is the absolute dielectric permittivity of the film, and *C*_el_ is the concentration of the electrolytes in m^−3^. We consider only symmetric 1:1 electrolytes. The film is aqueous, and the hydrophobic phase is insulating. Let the surfactant be anionic (e.g., sodium decyl sulfate, NaDS), of a concentration *C*_s_ = $C_{{\mathrm{DS}}^{-}}$, where *Φ* > 1 (i.e., the surface potential is negative). Let the background electrolyte be sodium chloride with a concentration C_NaCl_. In this case, it is valid that

(3) $$C_{\mathrm{el}}=C_{s}+C_{\mathrm{NaCl}}=C_{{\mathrm{DS}}^{-}}+C_{{\mathrm{Cl}}^{-}}=C_{{\mathrm{Na}}^{+}}$$

The Poisson–Boltzmann equation has serious limitations. For example it does not account for the ever-present ion-specific interactions and image forces [23], and the surface normal dipole moment (conjugated with the quadrupolarizabilities of the two phases) [24]. However, it is sufficient for a first analysis; besides, the Davies isotherm we use for the surfactant [25] is based on the same equation anyway.

Using the relation d^2^*ϕ*/d*z*^2^ = ½ d*E*^2^/d*ϕ*, we obtain a first integral of the Poisson–Boltzmann in Equations (1) and (2):

(4) $$\frac{\varepsilon }{2}E^{2}=-\int_{\phi_{m}}^{\phi }\rho d\phi =kTC_{\mathrm{el}}\frac{\left(\Phi -\Phi_{m}\right)\left(\Phi -\Phi_{m}^{-1}\right)}{\Phi },$$

where *Φ*_m_ = exp(−*eϕ*_m_/*kT*) and *ϕ*_m_ is the extremum of the potential (where *E* = 0—see Figure 1). The square root of Equation (4) yields:

(5) $$\frac{d\phi }{dz}=\pm \sqrt{\frac{2kTC_{\mathrm{el}}}{\varepsilon }\frac{\left(\Phi -\Phi_{m}\right)\left(\Phi -\Phi_{m}^{-1}\right)}{\Phi }}.$$

For negatively charged surfaces, *ϕ* is maximal in the center of the film. Therefore, for *z* < 0, the positive sign should be taken, and vice versa. Separation of variables and integration leads to:

(6) $$z=L_{D}\int_{\Phi_{m}}^{\Phi }\frac{d\Phi }{\sqrt{\Phi \left(\Phi -\Phi_{m}\right)\left(\Phi -\Phi_{m}^{-1}\right)}}=\frac{4L_{D}}{\sqrt{\Phi_{m}}}F\left(\sqrt{\frac{\Phi -\Phi_{m}}{\Phi -\Phi_{m}^{-1}}},\Phi_{m}^{-1}\right).$$

Here, *L*_D_ is the Debye length ($L_{D}^{2}$ = *εkT*/2*e*^2^*C*_el_), and F is the elliptic F integral. Integration from the center of the film (*z* = 0, *ϕ* = *ϕ*_m_) to one of the surfaces (*z* = *h*/2, *ϕ* = *ϕ*^S^) leads to a relation between the film thickness *h* and surface potential *ϕ*^S^:

(7) $$h=2L_{D}\int_{\Phi_{m}}^{\Phi^{S}}\frac{d\Phi }{\sqrt{\Phi \left(\Phi -\Phi_{m}\right)\left(\Phi -\Phi_{m}^{-1}\right)}}=\frac{4L_{D}}{\sqrt{\Phi_{m}}}F\left(\sqrt{\frac{\Phi^{S}-\Phi_{m}}{\Phi^{S}-\Phi_{m}^{-1}}},\Phi_{m}^{-1}\right),$$

where *Φ*^S^ = exp(−*eϕ*^S^/*kT*) is the surface Boltzmann factor. This equation can be solved for *Φ*^S^:

(8) $$\Phi^{S}=\Phi_{m}^{-1}+\frac{\Phi_{m}-\Phi_{m}^{-1}}{{\mathrm{cn}}^{2}\left(\frac{h\sqrt{\Phi_{m}}}{4L_{D}},\Phi_{m}^{-1}\right)},$$

where cn is the Jacobi cn function.

The Gauss condition relates the displacement field at the surface to the surface charge density *ρ*^S^:

(9) $$\varepsilon {d\phi /dz|}_{z=h/2}=\rho^{S}.$$

If the surface active species is an anion, then *ρ*^S^ is related to the adsorption *Γ* in the adsorption layer by the equation:

(10) $$\rho^{S}=-e\Gamma .$$

By substituting this and the Gauss Equation (9) into the first integral, Equation (4), one obtains the *generalized Gouy equation* which accounts for the confinement:

(11) $$\frac{L_{B}\Gamma^{2}}{2C_{\mathrm{el}}}=\frac{\left(\Phi^{S}-\Phi_{m}\right)\left(\Phi^{S}-\Phi_{m}^{-1}\right)}{\Phi^{S}};$$

here, *L*_B_ = *e*^2^/*εkT* is the Bjerrum length (without the usual factor 4π for more concise formulae). For a very thick film (*h* >> *L*_D_), it holds true that *ϕ*_m_ = 0 and *Φ*_m_ = 1 so that:

(12) $$\frac{L_{B}\Gamma^{2}}{2C_{\mathrm{el}}}=\Phi^{S}+{\left(\Phi^{S}\right)}^{-1}-2,$$

which is Gouy’s equation [26].

The dependence of the potential *ϕ* on *z* at a certain value of *Φ*_m_ is determined by Equation (6), and *Φ*_m_ itself follows from the values of *Γ* and *Φ*^S^ through Gouy’s generalized Equation (11). The values of *Γ* and *Φ*^S^ are determined by the boundary condition—Equation (7), and an adsorption isotherm, which we will consider next. The dependence of *ϕ* on *z* can already be illustrated for a chosen value of *Φ*_m_. A schematic example is given in Figure 1.

### 2.2. The Adsorption Isotherm

The condition for chemical equilibrium between the dissolved surfactant and the charged adsorption layer reads [3,27]:

(13) $$\gamma^{S}\Gamma =K_{a}C_{s}/\Phi^{S},$$

where *K*_a_ [m] is the adsorption constant, *C*_s_ is the concentration of surfactant, and *γ*^S^ is the surface activity coefficient, taking into account the lateral interactions between the adsorbed surface active ions. If |*eϕ*^S^/*kT*| ~ |ln*K*_a_*C*_s_| >> |ln*γ*^S^*Γ*|, then the condition predicts constant surface potential. If |*eϕ*^S^/*kT*| << |ln*K*_a_*C*_s_| ~ |ln*γ*^S^*Γ*|, then the surface charge is constant (true for densely packed monolayers in the presence of excess electrolytes). Otherwise, Equation (13) is a charge regulation condition.

The case of *γ*^S^ = 1 corresponds to “Henry’s” equation of the ionic surfactant:

(14) $$\Gamma =K_{a}C_{s}/\Phi^{S}.$$

Strictly speaking, Equation (14) is not Henry’s isotherm as it accounts for the main interaction in the charged monolayer, charge-charge, through *Φ*^S^ (i.e., it is at the nonlinear Debye–Hückel level of description). Davies [25,28] combined Equation (14) with Gouy’s Equation (12) under the assumption that *Φ*^S^ is an extremely large quantity (which is almost always fulfilled for a monolayer of an ionic surfactant):

(15) $$L_{B}\Gamma^{2}/2C_{\mathrm{el}}=\Phi^{S}.$$

By multiplying Equations (14) and (15) and solving them, we obtain for *Γ* the Davies isotherm on a single surface:

(16) $$\Gamma_{\infty }={\left(\frac{2K_{a}}{L_{B}}\right)}^{1/3}{\left(C_{s}C_{\mathrm{el}}\right)}^{1/3}.$$

Here, ∞ indicates that this adsorption is on an isolated surface (very thick film, *h* → ∞). By substituting this expression back into Equation (15), we obtain the Davies equation for the surface potential (surface Boltzmann factor) of a single surface:

(17) $$\Phi_{\infty }^{S}=\frac{L_{B}^{1/3}K_{a}^{2/3}}{2^{1/3}}\frac{C_{s}^{2/3}}{C_{\mathrm{el}}^{1/3}}.$$

Equations (16) and (17) work rather well for surfactants at W|O interfaces [29] in cases where the surface coverage is not too high (such that *γ*^S^ remains close to one).

Water|gas: isotherms with phase transition. In this case, a monolayer of an ionic surfactant typically exhibits two surface phases—2D gaseous and 2D liquid (known as *liquid expanded*)—with a phase transition taking place under specific conditions (concentration *C*_pt_, adsorption *Γ*_pt_ etc.). For many ionic surfactants, both phases follow the Davies isotherm but with a different adsorption constant [29]:

(18) $$\Gamma^{G}=K_{a}^{G}C_{s}/\Phi^{S}\;\mathrm{and}\;\Gamma^{\mathrm{LE}}=K_{a}^{\mathrm{LE}}C_{s}/\Phi^{S}.$$

It is always true that $K_{a}^{\mathrm{LE}}$ (the adsorption constant of the liquid expanded phase) is larger than $K_{a}^{G}$ (the true Henry’s constant); otherwise, only the gaseous phase is stable. For an isolated surface, the two equations in Equation (18) result in two Davies-like equations for *Γ* and *Φ*^S^ in the two phases, analogous to Equations (16) and (17) but with two different adsorption constants.

### 2.3. Disjoining Pressure

The local force balance in the film reads:

(19) ∇p=−ρ∇ϕ,

where *p* is the isotropic mechanical pressure. This is integrated using Equation (2):

(20) $$p=p_{0}+kTC_{\mathrm{el}}\left(\Phi +\Phi^{-1}-2\right),$$

where we treat *p* as a function of *ϕ*, and, therefore, *p*_0_ is the pressure which corresponds to zero potential *ϕ* (i.e., pressure in the meniscus or in a thick film). The respective pressure tensor ***P*** (including the Maxwell tensor) is given by [30]:

(21) $$𝘗=p𝚄-\varepsilon E^{2}e_{z}e_{z}+\frac{\varepsilon }{2}E^{2}𝚄.$$

Here, **U** is the unit tensor, and **e***_z_* is the unit vector in the *z* direction (normal to the film). This expression is approximate. For a draining film, the pressure tensor includes hydrodynamic terms [31]. When the surface carries a large dipole moment, and one of the phases is oil, the quadrupole terms of the Maxwell tensor also become important [32]. By substituting *p* from Equation (20) and *E*^2^ from Equation (4) in the expression for the pressure tensor, we obtain the following result for the normal component of ***P***:

(22) $$P_{zz}=p-\varepsilon E^{2}/2=p_{0}+\Pi_{\mathrm{el}},$$

where *Π*_el_ is, by definition [2], the electrostatic disjoining pressure in the film:

(23) $$\Pi_{\mathrm{el}}=kTC_{\mathrm{el}}\left(\Phi_{m}+\Phi_{m}^{-1}-2\right).$$

This result was first obtained by Langmuir [33], who treated the right-hand side of this equation as the change in osmotic pressure in the center of the film, *kTC*_el_(*Φ*_m_ + 1/*Φ*_m_), in comparison to the one in the meniscus, 2*kTC*_el_.

Under the constant potential regime, the surface potential is the same as that for a single isolated surface (subscript ∞), and thus $\Phi^{S}=\Phi_{\infty }^{S}$. In this case, Equations (7) and (23) define *Π*_el_ and *Φ*_m_, respectively, as functions of *h* (parametrically with parameter *Φ*_m_).

Under the constant charge regime, it is the surfactant adsorption which is constant (*Γ* = *Γ*_∞_), while the surface potential changes with *h*. In this case, Equations (7) and (23) and the generalized Gouy Equation (11) (where *Γ* is treated as a constant) determine *Π*_el_, *Φ*^S^, and *Φ*_m_ as functions of *h*.

In general, a fourth equation is required to determine *Π*_el_, *Φ*^S^, *Γ*, and *Φ*_m_, and this is the charge regulation condition. For this, we use the Davies isotherm for W|O, Equation (14), or two Davies isotherms with a phase transition for W|G, Equation (18).

For W|O (i.e., emulsion film), the electrostatic component given by Equation (23) dominates the disjoining pressure (the Hamaker constant for oil–water–oil is small [17]). For W|A (foam film), we must also add the Van der Waals interaction [2]:

(24) $$\Pi =kTC_{\mathrm{el}}\left(\Phi_{m}+\Phi_{m}^{-1}-2\right)-\frac{A_{H}}{6\pi }\frac{1}{h^{3}}.$$

Here, *A*_H_ is the Hamaker constant for water.

Let us also write down the respective formulae for the membrane tension of the film. This is given by the sum of the surface tensions *σ*^AL^ of the adsorbed layers, the contributions from the electric double layer ($\sigma_{m}^{\mathrm{DL}}$), and the Van der Waals interaction ($\sigma_{m}^{\mathrm{VdW}}$):

(25) $$\sigma_{m}=2\sigma^{\mathrm{AL}}+\sigma_{m}^{\mathrm{DL}}+\sigma_{m}^{\mathrm{VdW}}.$$

These components are calculated as follows. For *σ*^AL^, Henry’s equation holds true:

(26) $$\sigma^{\mathrm{AL}}=\sigma_{0}-kT\Gamma .$$

Here, *σ*_0_ is the interfacial tension of the surfactant-free interface, and Equation (26) is a form of the Davies isotherm [25]. The contribution of the electric double layer follows from the Maxwell tensor and the definition of membrane tension [2]:

(27) $$\sigma_{m}^{\mathrm{DL}}=2\int_{0}^{h/2}\left(P_{xx}-P_{zz}\right)dz.$$

Finally, for the Van der Waals contribution in foam films, we use the expression [17]:

(28) $$\sigma_{m}^{\mathrm{VdW}}=-\frac{A_{H}}{12\pi }\frac{1}{h^{2}}.$$

Equation (28) gives the free energy of the Van der Waals interaction between the two films.

For the liquid expanded state of the monolayer, Equation (26) must be modified to account for Langmuir’s liquid expanded oil-like film of hydrocarbon chains:

(29) $$\sigma^{\mathrm{AL}}=\sigma_{0}^{\mathrm{LE}}-kT\Gamma =\sigma_{0}+\pi_{\mathrm{coh}}-kT\Gamma ,$$

where *π*_coh_ is Langmuir’s cohesive pressure, a characteristic of the lateral interaction between the hydrocarbon tails in the liquid expanded film [29,34].

### 2.4. Charge Regulation According to the Davies Isotherm Versus Constant Charge or Potential

Constant potential. In this case, we assume that *Φ*^S^ is independent of *h*; it instead remains constant equal to $\Phi_{\infty }^{S}$, as for an isolated surface. The dependence of *Φ*_m_ on *h* is determined by Equation (7) (with $\Phi^{S}=\Phi_{\infty }^{S}$). The dependence of *Π*_el_ on *h* is determined parametrically by Equations (7) and (23), with the parameter *Φ*_m_ varying from 1 at *h* = ∞ to $\Phi_{\infty }^{S}$ at *h* = 0. When the potential is fixed, the adsorption of the surfactant changes with the film thickness. This *Γ*(*h*) dependence is determined parametrically by Equation (7) and Gouy’s Equation (11).

Such conditions are possible for electrode surfaces, where the potential is maintained by a potentiostat. However, for foam and emulsion films, once the film thickness *h* approaches the Debye length, the surface potential always changes significantly.

For the sake of comparison with the other regimes, let us give the asymptotes of the main characteristics for thick and thin films. The analysis which follows is equivalent to that of Honig and Mul [35]. We additionally assume that the surface potential is high (*Φ*^S^ >> 1) to fulfil the validity of the Davies isotherm.

For a thick film (*h* >> *L*_D_), the potential in the center of the film is close to zero, and *Φ*_m_ → 1. Here, the following well-known asymptote [2,33] holds true:

(30) $$\Pi_{\mathrm{el}}\overset{h\to \infty }{\to }64kTC_{\mathrm{el}}\gamma^{2}e^{-h/L_{D}},$$

where *γ* = tanh(−*eϕ*^S^/4*kT*).

At high potentials, *γ* = 1, and thus the disjoining pressure is independent of the surface potential.

In the other limiting case of an extremely thin film (*h* << *L*_D_), the electrostatic potential across the film is approximately constant, and one can use *Φ*^S^ → *Φ*_m_, which simplifies Equation (7) to:

(31) $$h\overset{\Phi^{S}\to \Phi_{m}}{\to }\frac{4L_{D}\sqrt{\Phi^{S}-\Phi_{m}}}{\sqrt{\Phi_{m}^{2}-1}}.$$

This asymptote can be inverted to the following parabolic dependence:

(32) $$\Phi_{m}=\Phi^{S}-\frac{h^{2}}{16L_{D}^{2}}{\left(\Phi^{S}\right)}^{2}.$$

**Figure 2:**
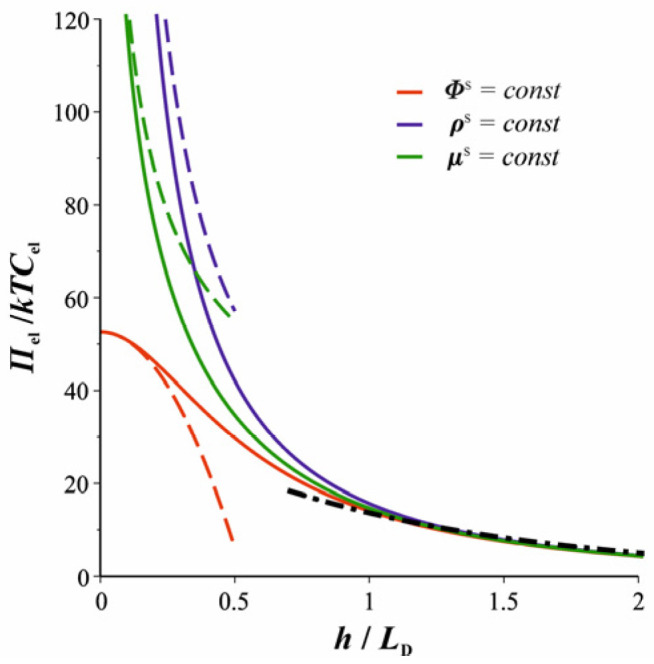
Comparison between the electrostatic component *Π*_el_ of the disjoining pressure as a function of the film thickness *h* (solid curves) for the three studied regimes: constant surface potential, charge, or chemical potential (see the text). The asymptote for the thick film (Equation (30)) is shown as a dashed dotted line. The asymptotes for thin films are given for the three regimes (dashed lines, Equations (33), (38), and (45)). All curves correspond to $\Phi_{\infty }^{S}$ = exp(+4) for the isolated surface. The curves depend on *C*_s_, *C*_el_, and *K*_a_ only through $\Phi_{\infty }^{S}$ (see Equation (17)).

Then, the respective disjoining pressure asymptote follows from Equation (23):

(33) $$\Pi_{\mathrm{el}}\overset{h\to 0}{\underset{\Phi^{S}=const}{\to }}kTC_{\mathrm{el}}\left[\Phi^{S}-\frac{h^{2}}{16L_{D}^{2}}{\left(\Phi^{S}\right)}^{2}\right],$$

where *Φ*^S^ >> 1 was again assumed. A comparison between the two asymptotes, Equations (30) and (33), and the exact parametric dependence of *Π*_el_ on *h* is illustrated in Figure 2.

Constant charge. The results again coincide with those of Honig and Mul [35] at *Φ*^S^ >> 1. In this case, we assume that the adsorption remains independent of the film thickness (i.e., *Γ* is equal to *Γ*_∞_) of the isolated surface. The surface potential, on the other hand, changes with *h.* From Gouy’s Equation (11), it follows that a constant charge corresponds to:

(34) $$\Phi^{S}+{\left(\Phi^{S}\right)}^{-1}-\Phi_{m}-{\left(\Phi_{m}\right)}^{-1}=\Phi_{\infty }^{S}+{\left(\Phi_{\infty }^{S}\right)}^{-1}-2,$$

where $\Phi_{\infty }^{S}$ refers to the isolated surface. By solving Equation (34) for *Φ*^S^ and substituting the solution in Equation (7) for *h*, we obtain *h* as a function of *Φ*_m_ for the constant *Γ*. We then obtain *Π*_el_(*h*) parametrically from *h*(*Φ*_m_) and *Π*_el_(*Φ*_m_) via Equation (23).

The asymptote for thick films still follows Equation (30). For *h* < *L*_D_, however, the behavior is quite different for the two regimes. For a thin film, all potentials are very high, and *Φ* >> 1, irrespective of the indices. Therefore, Equation (34) simplifies to

(35) $$\Phi_{m}=\Phi^{S}-\Phi_{\infty }^{S}.$$

On the other hand, we can expand Equation (8) in series:

(36) $$\Phi^{S}\overset{h\to 0}{\underset{\Gamma =const}{\to }}\Phi_{m}+\frac{h^{2}}{16L_{D}^{2}}\Phi_{m}^{2}.$$

Combining the last two equations leads to an explicit asymptote for *Φ*_m_(*h*):

(37) $$\Phi_{m}\overset{h\to 0}{\underset{\Gamma =const}{\to }}\frac{4L_{D}\sqrt{\Phi_{\infty }^{S}}}{h}.$$

By substituting this *Φ*_m_ into Equation (23) for the disjoining pressure, we obtain the respective asymptote of *Π*_el_:

(38) $$\Pi_{\mathrm{el}}\overset{h\to 0}{\underset{\Gamma =const}{\to }}kTC_{\mathrm{el}}\left(\frac{4L_{D}\sqrt{\Phi_{\infty }^{S}}}{h}+\frac{h}{4L_{D}\sqrt{\Phi_{\infty }^{S}}}-2\right)\approx kTC_{\mathrm{el}}\left(\frac{4L_{D}\sqrt{\Phi_{\infty }^{S}}}{h}-2\right).$$

This is compared to the exact solution in Figure 2. The exact dependence of *Π*_el_ versus *h* under a constant charge regime is calculated via Equations (23) and (7) and the generalized Gouy Equation (11) (where *Γ* is treated as a constant equal to *Γ*_∞_), which determine *Π*_el_, *Φ*^S^, and *Φ*_m_ as functions of *h*. This is realized numerically by solving Equation (11) for *Φ*^S^, substituting this *Φ*^S^ into Equation (7), and plotting *Π*_el_(*Φ*_m_) from Equation (23) against *h*(*Φ*_m_) from Equation (7) parametrically.

Constant chemical potential (charge regulation regime). In reality, the thinning of emulsion and foam films is under a constant chemical potential, and both *Γ* and *Φ*^S^ change with the thickness. To determine the electrostatic disjoining pressure *Π*_el_ for this case, we first substitute *Γ* from the isotherm, Equation (14), into the generalized Gouy Equation (11):

(39) $$\frac{L_{B}}{2C_{\mathrm{el}}}\frac{K_{a}^{2}C_{s}^{2}}{{\left(\Phi^{S}\right)}^{2}}=\Phi^{S}+{\left(\Phi^{S}\right)}^{-1}-\Phi_{m}-{\left(\Phi_{m}\right)}^{-1}.$$

For an isolated surface (or extremely thick film of *Φ*_m_ = 1), the above equation simplifies to:

(40) $$\frac{L_{B}}{2C_{\mathrm{el}}}\frac{K_{a}^{2}C_{s}^{2}}{{\left(\Phi_{\infty }^{S}\right)}^{2}}=\Phi_{\infty }^{S}+{\left(\Phi_{\infty }^{S}\right)}^{-1}-2.$$

The last two equations can be combined to obtain the following convenient form:

(41) $$\frac{\Phi_{\infty }^{S}{\left(\Phi_{\infty }^{S}-1\right)}^{2}}{{\left(\Phi^{S}\right)}^{2}}=\Phi^{S}+{\left(\Phi^{S}\right)}^{-1}-\Phi_{m}-{\left(\Phi_{m}\right)}^{-1}.$$

We solve Equation (41) for *Φ*^S^ and then we substitute the result into Equation (7) to obtain *h*(*Φ*_m_). This determines *Π*_el_(*h*) parametrically via this *h*(*Φ*_m_) and *Π*_el_(*Φ*_m_) from Equation (23).

The asymptote at a large thickness is again unchanged compared to Equation (30). To analyze the asymptote for a thin film (*h* << *L*_D_), we assume that all Boltzmann factors are very large, which allows us to simplify Equation (41) to:

(42) $$\Phi^{S}-\Phi_{m}\approx \frac{{\left(\Phi_{\infty }^{S}\right)}^{3}}{{\left(\Phi^{S}\right)}^{2}}$$

The respective asymptote of *h* from Equation (7) reads as follows:

(43) $$h\approx \frac{4L_{D}}{\sqrt{\Phi_{m}^{2}-1}}\frac{{\left(\Phi_{\infty }^{S}\right)}^{3/2}}{\Phi^{S}}\approx \frac{4L_{D}}{\Phi_{m}}\frac{{\left(\Phi_{\infty }^{S}\right)}^{3/2}}{\Phi^{S}}$$

Since for a thin film *Φ*^S^ ≈ *Φ*_m_, we can write the last equation as:

(44) $$\Phi_{m}\approx \frac{2\sqrt{L_{D}}{\left(\Phi_{\infty }^{S}\right)}^{3/4}}{\sqrt{h}}$$

This result is substituted into Langmuir’s Equation (23) for *Π*_el_:

(45) $$\frac{\Pi_{\mathrm{el}}}{kTC_{\mathrm{el}}}\overset{h\to 0}{\underset{\mu^{S}=const}{\to }}\frac{2\sqrt{L_{D}}{\left(\Phi_{\infty }^{S}\right)}^{3/4}}{\sqrt{h}}+\frac{\sqrt{h}}{2\sqrt{L_{D}}{\left(\Phi_{\infty }^{S}\right)}^{3/4}}-2\approx \frac{2\sqrt{L_{D}}{\left(\Phi_{\infty }^{S}\right)}^{3/4}}{\sqrt{h}}-2$$

Thus, the disjoining pressure of a fluid film stabilized by an ionic surfactant follows an *h*^−1/2^ asymptote at small thicknesses. It depends solely on the surface potential of the thick film; two surfactant monolayers of the same surface potential will produce the same asymptote irrespective of the actual value of the adsorption constant *K*_a_.

Let us, for completeness, also write down the asymptotes of the surface potential and the surface charge at small *h* values:

(46) $$\Phi^{S}\overset{h\to 0}{\underset{\mu^{S}=const}{\to }}\frac{2\sqrt{L_{D}}}{\sqrt{h}}{\left(\Phi_{\infty }^{S}\right)}^{3/4}+\frac{h}{4L_{D}}{\left(\Phi_{\infty }^{S}\right)}^{3/2}$$

(47) $$\Gamma \overset{h\to 0}{\underset{\mu^{S}=const}{\to }}\sqrt{\frac{C_{\mathrm{el}}h}{2L_{D}L_{B}}}{\left(\Phi_{\infty }^{S}\right)}^{3/4}.$$

In all of these formulae, $\Phi_{\infty }^{S}$ is given by the Davies Equation (17).

The results from this section are general in the sense that they account for the presence of background electrolytes and the ion-specific effect (through the counterion-specific value of the adsorption constant *K*_a_ [29]). They have, however, the same limitations as the Davies isotherm; they hold for coverages of up to 30–60%, depending on the surfactant. Typically, monolayers of ionic surfactants alone are not particularly dense anyway.

### 2.5. Phase Transition at W|A Interface Triggered by Film Thinning

The previous section is most relevant to emulsion films stabilized by soluble ionic surfactants, where the Davies isotherm is a particularly good model [29]. What makes a foam film different is the large cohesion between the hydrocarbon tails of the adsorbed surfactant, which produces $K_{a}^{\mathrm{LE}}$ in Equation (18) and *π*_coh_ in Equation (29). We will now consider a foam film covered with monolayers close to the phase transition point between the 2D gaseous and liquid expanded states. For an isolated surface, the phase transition point corresponds to equal surface tension of the two phases (i.e., *σ* = *σ*^AL^ + *σ*^DL^ is the same for the two phases), where for an isolated surface, *σ*^AL^ is given by Equation (26) or Equation (29), and *σ*^DL^ = 2*kTΓ* [25,29]. Therefore, the Davies isotherms for the two phases give for the surface pressures of the two phases:

(48) $$\pi^{G}\equiv \sigma_{0}-\sigma^{G}=3kT\Gamma^{G}\;\mathrm{and}\;\pi^{\mathrm{LE}}\equiv \sigma_{0}-\sigma^{\mathrm{LE}}=-\pi_{\mathrm{coh}}+3kT\Gamma^{\mathrm{LE}},$$

with *Γ* given by Equation (16), with two different *K*_a_ values for the two phases. The phase transition condition reads:

(49) $$\pi_{\mathrm{pt}\infty }=3kT\Gamma^{G}=-\pi_{\mathrm{coh}}+3kT\Gamma^{\mathrm{LE}}.$$

This equation determines the phase transition point for an isolated surface.

We determined the three parameters of the W|A isotherm—$K_{a}^{G}$, $K_{a}^{\mathrm{LE}}$, and *π*_coh_—by comparing the Davies isotherms with tensiometric data for various surfactants, which were limited to *π*^S^ < 10 mM to make sure that the condition *γ*^S^ = 1 is fulfilled. The procedure for determination of the parameters was like the one in [29]. The data for C_10_H_21_OC_2_H_4_SO_3_Na were from [36]. The data for C_12_H_25_SO_4_Na were from [37,38,39,40]; the data for C_10_H_21_N(CH_3_)_3_Br—from [41]; for C_12_H_25_N(CH_3_)_3_Br—from [42]; for C_12_H_25_N(CH_3_)_3_Cl —from [43]. The adsorption parameters are listed in Table 1.

In some cases (C_10_H_21_OC_2_H_4_SO_3_Na), data for the gaseous phase were not available. It is often the case that even the lowest concentration studied experimentally is already in the liquid expanded state, as researchers are rarely interested in regions of low surface pressure. We therefore recalculated $K_{a}^{G}$ for the surfactant C_10_H_21_OC_2_H_4_SO_3_Na using the value of $K_{a}^{G}$ for C_12_H_25_SO_4_Na, combined with Traube’s rule, as described in [29].

With the parameters from Table 1, we determined the phase transition point by solving Equation (49) for *C*_s_ and the two adsorptions given by the Davies isotherm, Equation (16). We assumed that no background electrolyte was present, and therefore, *C*_el_ = *C*_s_. This leads to:

(50) $${\left(C_{s,\mathrm{pt}\infty }\right)}^{2/3}=\frac{\pi_{\mathrm{coh}}}{3kT\left[{\left(2K_{a}^{\mathrm{LE}}/L_{B}\right)}^{1/3}-{\left(2K_{a}^{G}/L_{B}\right)}^{1/3}\right]}.$$

The respective surface pressure at the phase transition for the isolated surface follows from Equation (49).

**Figure 3:**
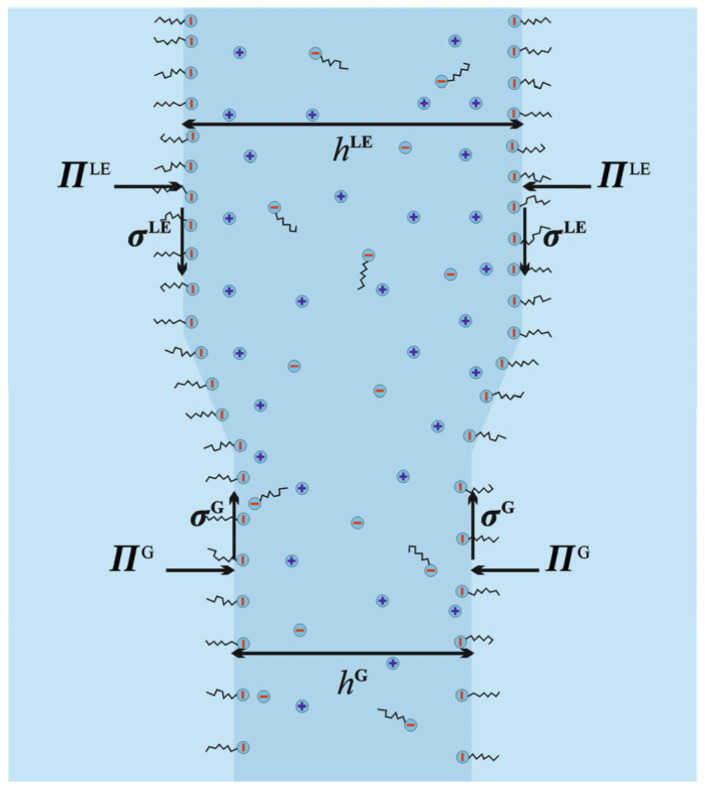
Illustration of phase transition in air–water–air thin liquid film.

The phase transition in a foam film occurs at a slightly higher concentration compared to an isolated surface (i.e., *C*_s_ > *C*_s,pt∞_). For example, if we make a soap film from a solution of *C*_s_ = 0.85 mM sodium dodecyl sulfate, then the two isolated surfaces in a thick film will be in the liquid expanded state (since *C*_s,pt∞_ = 0.81 mM). However, as the film thins, the interactions between the two surfaces rarefy the monolayer, and eventually, a critical transition thickness is reached where LE is no longer the stable phase. At this point, the film is expected to make a phase transition to a 2D gaseous state, which is simultaneously of a smaller surface charge and surface potential and therefore thinner compared with the LE film. In Sheludko’s interferometric cell, this should be observed as nucleation and growth of a darker (i.e., thinner) spot of a film covered with a gaseous monolayer within the initial lighter (thicker) film covered with a liquid expanded monolayer. The geometry of such a heterogeneous film is illustrated in Figure 3. Similar spot formations are often observed in films transiting from common films (electrostatically stabilized) to Newton black films (sterically stabilized) [44]. The phenomenon has also been observed in micellar solutions, which tend to make phase transitions through stepwise expulsion of micellar layers [45]. To our knowledge, such spot formations have never been observed for the proper surface phase transition discussed here.

**Figure 4:**
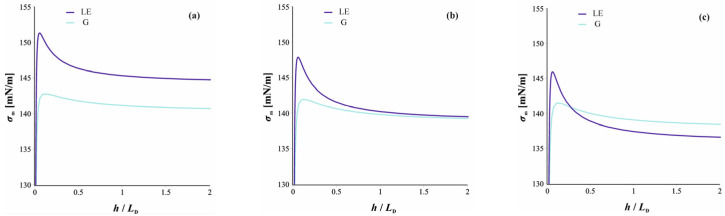
Membrane tension as a function of the film thickness (nondimensionalized with the Debey length *L*_D_). The parameters are for sodium dodecyl sulfate. The three figures correspond to three concentrations: (**a**) *C*_s_ = 0.50 mM, which is below the phase transition of the isolated surface; (**b**) *C*_s_ = 0.81 mM, which is at the phase transition; (**c**) *C*_s_ = 1.00 mM, which is above the phase transition. The blue curve stands for film covered with a gaseous monolayer, and the purple is for the LE phase.

Let us now predict the actual thicknesses at which the phase transition should be observed. The condition for chemical equilibrium was automatically fulfilled as we fixed the chemical potentials of the surfactant to that in the meniscus through the generalized Davies isotherm. The condition for mechanical equilibrium between the two films—the thicker LE and the thinner LG—requires that the membrane tensions of the two films are equal (tangential force balance) and the disjoining pressures are also equal (normal balance). The membrane tensions $\sigma_{m}^{\mathrm{LE}}$ and $\sigma_{m}^{G}$ are given by Equation (25) (with the contributions from the adsorption layer, the electric double layer, and the Van der Waals interaction given by Equations (26)–(28)). These are illustrated as functions of *h* in Figure 4.

**Figure 5:**
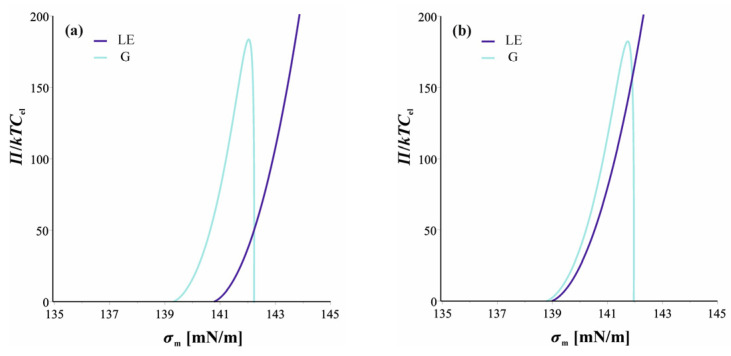

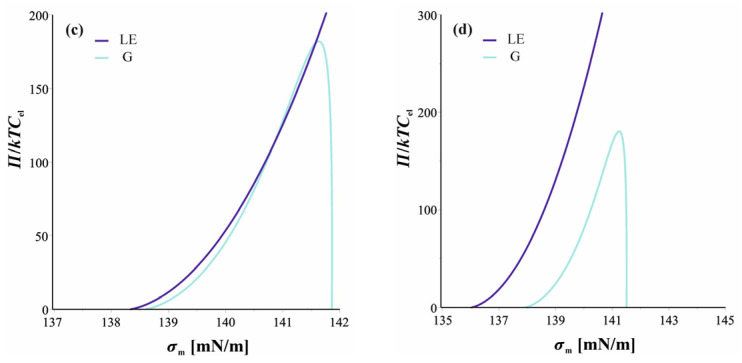
Determination of the point of phase transition triggered by film drainage for films stabilized with sodium dodecyl sulfate, with disjoining pressure *Π* versus membrane pressure *σ*_m_. (**a**) *C*_s_ = 0.70 mM. There is a crossing point, but it corresponds to unstable equilibrium. (**b**) *C*_s_ = 0.81 mM (equal to *C*_s,pt∞_ of the isolated surface). Again, there is unstable equilibrium. (**c**) *C*_s_ = 0.85 mM. In this case, the crossing point near *Π* = 100*kTC*_el_ corresponds to two films in stable equilibrium with two different thicknesses: *h*^G^ = 4.9 nm and *h*^LE^ = 6.2 nm. (**d**) *C*_s_ = 1.0 mM. No crossing point – no phase transition is possible.

To determine the point of the phase transition, we solve the two conditions for mechanical balance simultaneously. This can be illustrated graphically by plotting the disjoining pressure *Π*(*h*) from DLVO’s Equation (24) against *σ*_m_(*h*). This is shown in Figure 5 for four different concentrations of surfactants. The first concentration is below the phase transition point of the isolated surface (Figure 5a), and the second is exactly at the phase transition point (Figure 5b). In both cases, the two curves for the gaseous and LE films cross each other, but the crossing point appears at thicknesses where the films are unstable, being below the maximum of *σ*_m_ (Figure 4), where they thin until breakage. Therefore, films stabilized by a monolayer in a 2D gaseous state remain in the gaseous state without a phase transition.

The behavior is different for *C*_s_ = 0.85 mM, slightly higher than the concentration at the phase transition. In this case, the *Π* versus *σ*_m_ curves cross twice (see Figure 5c). One of the crossing points (which appears at higher *Π* values) corresponds to an unstable state again. The other one, however, corresponds to a stable equilibrium, with the two films coexisting as shown in Figure 3. We determined the crossing point numerically as values for *Π*_pt_ and *σ*_m,pt_. From the obtained value of *σ*_m,pt_, we calculated the thicknesses of the gaseous film and the LE film from curves like those in Figure 4. The thicknesses obtained this way are listed for a couple of surfactant concentrations in the meniscus in Table 1.

Finally, when the concentration of surfactants is much higher than *C*_pt_, the two curves for the gaseous film and LE film do not cross each other (Figure 5d). Therefore, the film thinning cannot trigger a phase transition, and the film thins until breakage, remaining in the liquid expanded state.

## 3. Conclusions

We analyzed the surface forces in thin liquid films stabilized by ionic surfactants which drain in the charge regulation regime. We used the simplest possible Davies isotherm as a charge regulation condition for two particular systems: emulsion and foam films.

We showed that for the oil–water–oil film, this charge regulation condition predicts the electrostatic disjoining pressure between the two classical constant charge and constant potential regimes (see Figure 2). We also derived the asymptotic behavior of *Π*_el_ at small thicknesses, and we showed that the Davies regulation results in *Π*_el_ being proportional to *h*^−1/2^, Equation (45).

We also investigated the thinning of a foam film stabilized by an ionic surfactant which forms a cohesive monolayer transiting from a 2D gaseous state to a liquid expanded state. We showed that it is in theory possible for such foam films to make a phase transition from a thicker film, covered with liquid expanded phase, to a thinner film covered with gaseous monolayers. We analyzed the concentration range where such a transition is possible. We showed it occurs in a tight region spanning from the phase transition concentration *C*_s,pt∞_ of an isolated surface to one that is 10–20% higher (and varies with the surfactant). The film thicknesses of the two films in equilibrium were calculated for a few cases (Table 1).

The intention of this article is to investigate for the first time the charge regulation in fluid films, foam or emulsion, and to provide estimates of the parameters of films thinning under this regime to guide future experimental work on the phenomenon. The results show that, for single surfactant systems, charge regulation becomes important at extremely high disjoining pressures. Such pressures will be difficult to achieve in a Sheludko cell, where the compressing pressure is limited to a few tens of kPa. The phenomenon will be easier to detect in films of lower charges stabilized by a mixture of ionic and nonionic surfactants (which is the most important case in practice anyway). However, the theory in this case is more complicated and beyond the objectives of our paper. Charge regulation will also be important for microemulsions or coalescence of extremely small droplets (radius ~1 µm).

We also predicted that the thinning of a foam film can trigger a surface phase transition. This phase transition should take place with a simultaneous jump in the film thickness. The observation of such a film phase transition will be similarly difficult in a Scheludko cell for a single ionic surfactant, but it should be possible with mixed monolayers or monolayers of protolytes which are only partially charged to reduce the electrostatic repulsion to values achievable in this apparatus. The phase transition should be expected to appear in the usual way, with the formation and growth of a dark spot within the lighter initial film. The dark spot will be stabilized by a 2D gaseous monolayer of a surface charge lower than that of the surrounding film covered by an LE monolayer. A similar phase transition can be expected slightly above the phase transition concentration *C*_pt∞_ for any two phases of a charged monolayer.

## Figures and Tables

**Table 1:** Phase transition parameters for foam films stabilized by various surfactants.

Surfactant	Adsorption Parameters			Phase Transition for Isolated Surface (mM, mN/m)	Phase Transition in a Film	
	ln($K_{a}^{G}$/m)	ln($K_{a}^{\mathrm{LE}}$/m)	*π*_coh_ [mN/m]			
					*h*^G^ [nm]	*h*^LE^ [nm]
C_10_H_21_OC_2_H_4_SO_3_Na	−10.31	−7.42	−3.1	*C*_s,pt∞_ = 1.18 mM *π*_pt∞_ = 1.9 mN/m	*C*_s_ = 1.20 mM	
					11.0	12.8
					*C*_s_ = 1.25 mM	
					5.2	6.9
C_12_H_25_SO_4_Na	−8.25	−4.64	−7.0	*C*_s,pt∞_ = 0.81 mM *π*_pt∞_ = 3.0 mN/m	*C*_s_ = 0.85 mM	
					4.9	6.2
					*C*_s_ = 1.00 mM	
					-	-
C_10_H_21_N(CH_3_)_3_Br	−11.62	−8.63	−3.5	*C*_s,pt∞_ = 2.51 mM *π*_pt∞_ = 2.1 mN/m	*C*_s_ = 2.53 mM	
					22.2	23.9
					*C*_s_ = 2.60 mM	
					7.3	8.9
C_12_H_25_N(CH_3_)_3_Br	−10.57	−5.74	−6.4	*C*_s,pt∞_ = 1.02 mM *π*_pt∞_ = 1.6 mN/m	*C*_s_ = 1.05 mM	
					7.2	9.9
					*C*_s_ = 1.10 mM	
					-	-
C_12_H_25_N(CH_3_)_3_Cl	−10.65	−7.27	−3.1	*C*_s,pt∞_ = 0.95 mM *π*_pt∞_ = 1.5 mN/m	*C*_s_ = 0.97 mM	
					15.2	17.6
					*C*_s_ = 1.00 mM	
					7.3	9.7

## Data Availability

Data are contained within the article.

## Abbreviations

*A*_H_	Hamaker constant
*C*_el_	total electrolyte concentration
*C*_s_	surfactant concentration
*C*_s,pt__∞_	phase transition concentration of an isolated surface
*E*	electric field
*e*	elementary electric charge
*K*_a_	equilibrium adsorption constant (from the aqueous phase)
$K_{a}^{G}$	adsorption constant for gaseous phase
$K_{a}^{\mathrm{LE}}$	adsorption constant for liquid expanded phase
*k*	Boltzmann constant
*L*_B_	Bjerumm’s length (*L*_B_ = *e*^2^/*εkT*)
*L*_D_	Debye’s length ($L_{D}^{2}$ = *εkT*/2*e*^2^*C*_el_)
***P***	pressure tensor (including the Maxwell tensor)
*p*	isotropic mechanical pressure
*T*	temperature
**U**	unit tensor
*z*	distance from the center of the film
*Γ*	adsorption of the surfactant
*Γ* ^G^	surfactant adsorption for 2D gaseous phase
*Γ* ^LE^	surfactant adsorption for liquid expanded phase
*γ* ^S^	surface activity coefficient of the surfactant
*ε*	absolute dielectric permittivity
*μ* ^S^	chemical potential of the surface active ion
*Π*	disjoining pressure
*Π*_el_	electrostatic disjoining pressure
*π*	surface pressure of the monolayer (*π* ≡ *σ*_0_ – *σ*)
*π*_coh_	Langmuir’s cohesive pressure
*π*_pt__∞_	phase transition surface pressure of an isolated surface
*π* ^G^	surface pressure for gaseous phase
*π* ^LE^	surface pressure for liquid expanded phase
*ρ*	charge density
*ρ* ^S^	surface charge (*ρ*^S^ = −*eΓ*) for ionic 1:1 surfactant
*σ*	surface tension of the monolayer
*σ*_0_	surface tension of the neat surface
*σ* ^AL^	contribution of the adsorbed layer to the surface tension
*σ*_m_	membrane tension
$\sigma_{m}^{\mathrm{DL}}$	contribution of the electric double layer to the membrane tension
$\sigma_{m}^{\mathrm{VdW}}$	Van der Waals contribution to the membrane tension
*Φ*	Boltzmann factor for a cation (*Φ* = exp(−*eϕ*/*kT*))
*Φ*_m_	Boltzmann factor in the center of the film (*Φ*_m_ = exp(−*eϕ*_m_/*kT*))
*Φ* ^S^	surface Boltzmann factor (*Φ*^S^ = exp(−*eϕ*^S^/*kT*))
*ϕ*	electrostatic potential
*ϕ*_m_	extremum of the electrostatic potential in the center of the film
*ϕ* ^S^	surface electric potential
2D	two-dimensional
*CMC*	critical micelle concentration
EoS	equation of state
W|G	water–gas surface
W|O	water–oil interface
